# ﻿Taxonomic study on the genus *Stenochironomus* Kieffer from the Baishanzu Nature Reserve, China (Diptera, Chironomidae)

**DOI:** 10.3897/zookeys.1104.81403

**Published:** 2022-06-09

**Authors:** Chao Song, Bin-Qing Zhu, Joel Moubayed-Breil, Teng Lei, Xin Qi

**Affiliations:** 1 College of Life Sciences, Taizhou University, Taizhou, Zhejiang 318000, China Taizhou University Taizhou China; 2 Nanjing Institute of Environmental Sciences, Ministry of Ecology and Environment, Nanjing 210042, Jiangsu, China Nanjing Institute of Environmental Sciences, Ministry of Ecology and Environment Nanjing China; 3 Freshwater & Marine biology, 10 rue des Fenouils, F-34070 Montpellier, France Freshwater & Marine biology Montpellier France

**Keywords:** Chironominae, DNA barcode, new species, non-biting midge, *Stenochironomus*, taxonomy

## Abstract

During the summer of July to September 2020, a biodiversity survey on Chironomidae of Baishanzu Nature Reserve, China was made. In total, five *Stenochironomus* taxa/species were discovered, of which two belong to undescribed species and one (*S.okialbus* Sasa, 1990) is reported for the first time from China. The male adults of two new species are described and illustrated. *Stenochironomusannulus* Song & Qi **sp. nov.** is distinguished in having a wing with two dark spots restricted to the fork area of FCu and RM, the mid- and hind-femur each with a brown annulus, and the inferior volsella with two setae and one strong terminal spine. *Stenochironomusbaishanzuensis* Song & Qi **sp. nov.** is distinguished by a combination of characters: a single dark spot on the middle part of the wing, fore legs brown to dark brown except for the basal 3/4 of femur, and the inferior volsella with four long setae and one stout terminal spine. The neighbour-joining tree based on public COI barcodes formed distinct clades with clear support for the new species. An updated key to known male adults of *Stenochironomus* from China is also provided.

## ﻿Introduction

The genus *Stenochironomus* Kieffer, 1919 has a cosmopolitan distribution in all zoogeographical regions except for Antarctica (Cranston et al. 1989). The larvae are known as miners and occur in different habitats such as dead wood, dead leaves, and floating leaves of lotuses, *Nelumbo* (Borkent, 1984). Male adults are characterized by a combination of characters as documented by Borkent (1984) and Townes (1945): different color patterns of the thorax and legs; short to elongate, variably sausage-shaped superior volsella with several bristles; and long narrow curved inferior volsella with few apical bristles and terminal spines. *Stenochironomus* is composed of two subgenera, *Stenochironomuss.str.* and *Petalopholeus*, which cannot be differentiated on imaginal morphology.

Data on the taxonomy, keys, and geographical distributions for *Stenochironomus* show that there are 110 known valid species recorded worldwide, of which 14 species are reported from China (Qi et al. 2008a, b, 2011, 2015; Dantas et al. 2016; Parise and Pinho 2016; Zhang et al. 2016; Amora et al. 2018; Lin et al. 2021).

DNA barcoding employs sequence diversity in short, standardized, gene regions and has become an important tool for species identification and cryptic species discovery (Hebert et al. 2003). Chironomid researchers also confirmed the effectiveness of a DNA barcode reference library in the discovery of new species using the 658-bp fragment of the mitochondrial gene cytochrome c oxidase I (COI) (Kodama et al. 2018; Lin et al. 2020, Qi et al. 2020; Song et al. 2020).

Baishanzu is a nature reserve, spanning the south Zhejiang and north Fujian provinces of eastern China. It belongs to the tropical to warm temperate transitional zone and is a biodiversity hot spot in Asia with the dominant types of vegetation being evergreen broad-leaved forests and mixed coniferous and broad-leaved forests (Peng et al. 2012). During seasonal surveys of the nature reserve, five *Stenochironomus* taxa/species were discovered, of which two belong to undescribed species and one, *S.okialbus* Sasa, 1990, is reported for the first time from China. In addition, DNA barcodes of the new species were analyzed and clearly supported them as new species. An updated key to male adults of known *Stenochironomus* from China is also provided.

## ﻿Materials and methods

### ﻿Morphological study

The examined material was collected using light traps; the specimens were preserved in 75% ethanol at 4 °C in a refrigerator before final slide mounting. Specimens were side-mounted in Euparal after genomic extraction following the procedure described by Sæther (1969). Morphological terminology follows that of Sæther (1980). The photographs of the habitus of each specimen were obtained with a DV500 5MP Digital Camera attached to a stereo microscope (Chongqing Optec SZ680). The photographs of the body parts were obtained using a Leica DMLS compound microscope. Photograph post-processing was done in Adobe Photoshop and Illustrator version 8 (Adobe Inc., California, USA).

The type material including holotype and paratypes of the two new described species are deposited in the collection of the College of Life Sciences, Taizhou University, Taizhou, China (TZU).

### ﻿Abbreviations used are as follows:

**AR** antennal ratio, length of the 13^th^ / length of flagellomeres 1–12;

**BV** length of (femur + tibia + ta_1_) / length of (ta_2_ + ta_3_ + ta_4_ + ta_5_);

**Cu** cubitus;

**Dc** dorsocentrals;

**Fe** femur;

**HR** hypopygium ratio, length of gonocoxite / length of gonostylus;

**HV** hypopygium value, total length / 10* length of gonostylus;

**IV** inner verticals;

**LR** leg ratio; Length of ta_1_ / length of tibia;

**M** media;

**MCu** cross-vein between media and cubitus;

**P1, P2, P3** Fore leg, mid leg, hind leg;

**R** radius;

**RM** cross-vein between radius and media;

**Ta**tarsomere;

**Ti** tibia;

**VR** venarum ratio, length of Cu / length of M.

### ﻿Molecular study

Tissues for total genomic DNA extraction were removed from the thorax and head of the adults. The genomic extraction procedure followed Frohlich et al. (1999). The standard barcode region of the 5’ portion of the mitochondrial gene cytochrome c oxidase I (COI-5P) was amplified using the universal primers LCO1490 and HCO2198 (Folmer et al. 1994); PCR amplifications followed Song et al. (2018). PCR products were electrophoresed in 1.0% agarose gel, purified, and sequenced using an ABI 3730XL capillary sequencer (Beijing Genomics Institute Co., Ltd., Hangzhou, China). Raw sequences were edited in BioEdit 7.2.5 (Hall 1999).

Public *Stenochironomus* sequences were searched in GenBank, and 32 sequences were returned, of which eleven sequence were mitochondrion complete genomes. We extracted COI-5P barcode segments from those genomes.

The pairwise distances were calculated using the Kimura 2-Parameter (K2P) substitution model in MEGA 7 (Kumar et al. 2016). The neighbour-joining tree was constructed using the K2P substitution model, and 1000 bootstrap replicates and the “complete deletion” option for missing data were utilized. Automatic Barcode Gap Discovery (ABGD) analysis was implemented on the website (wwwabi.snv.jussieu.fr/public/ abgd/abgdweb.html, Puillandre et al. 2012), with K2P model. Sequences, trace-files, and metadata of the new species were uploaded to the Barcode of Life Data Systems (BOLD) (Ratnasingham and Hebert 2013).

## ﻿Results and discussion

### ﻿Barcode analysis

**Figure 1.**
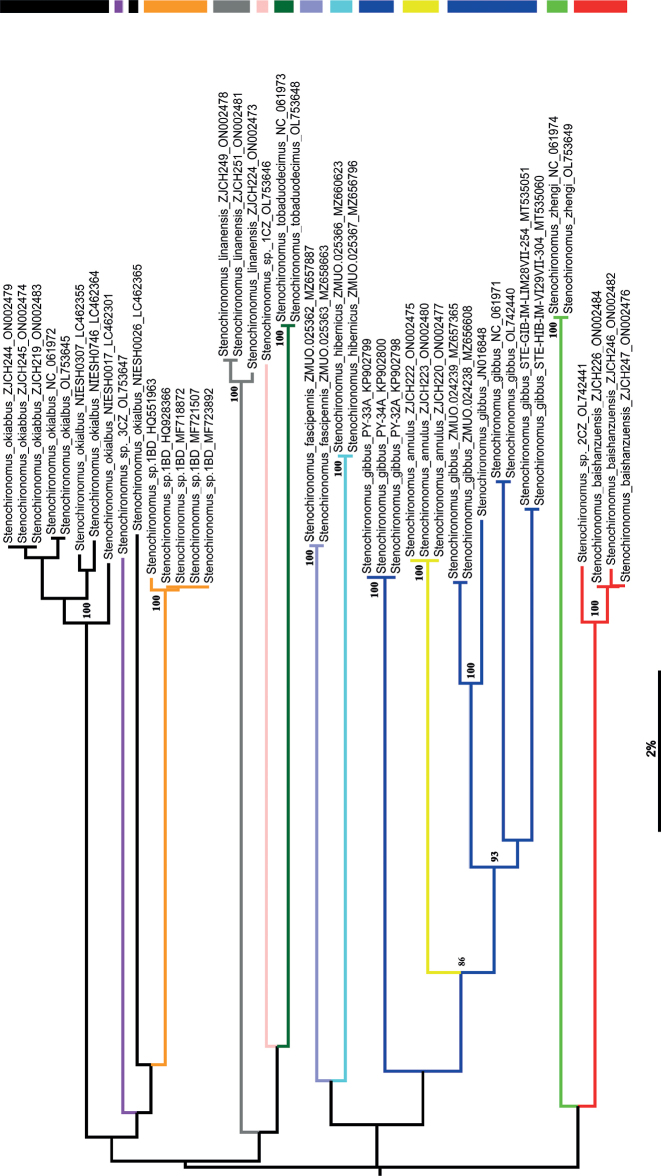
Neighbour-joining tree for twelve species of *Stenochironomus* based on K2P distance in DNA barcodes. Clade in yellow represents *S.annulus* sp. nov., red represents *S.baishanzuensis* sp. nov. Numbers on branches represent bootstrap support (>75%) based on 1000 replicates; scale equals K2P genetic distance.

**Figure 2.**
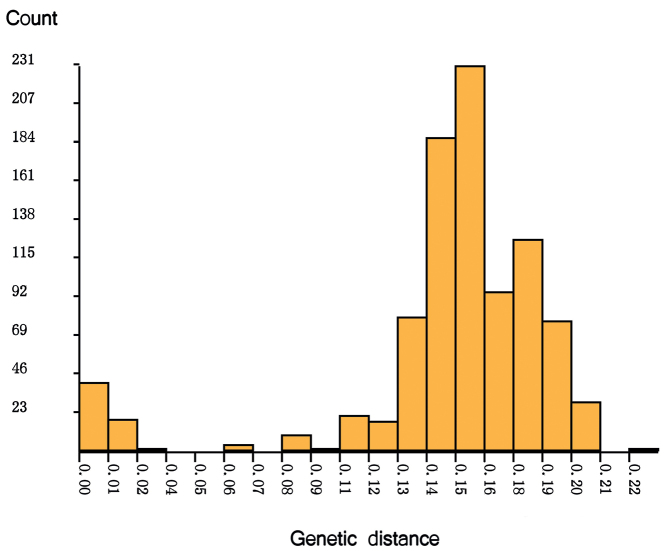
Histogram of pairwise K2P distances of public *Stenochironomus* sequences, generated by web site of ABGD.

All 44 public COI-5P DNA barcodes comprising GenBank accessions and sequences from this study (Table 1) representing 12 species within *Stenochironomus* were used to construct the neighbour-joining tree. The twelve species formed 16 distinct genetic clades; two clades separately presented for the new species *Stenochironomusannulus* sp. nov., and *S.baishanzuensis* sp. nov. (Fig. 1). *Stenochironomusannulus* sp. nov. can be distinguished from other species by more than 11.2%, and *S.baishanzuensis* sp. nov. by more than 14.0% (Table 2). In the barcoded *Stenochironomus* species, there is a gap between 4–6% (Fig. 2), which may be used for the delimitation of *Stenochironomus* species. The thresholds for different Chironomidae groups are not always the same; for example, Lin et al. (2015) found a gap of 4–5% for *Tanytarsus* and Song et al. (2016, 2018) found a gap of 5–8% for *Polypedilum*. However, the average genetic distance for *S.gibbus* is up to 9.1% (ranging from 0 to 13.0%), and for *S.okiabbus* is 3.95% (ranging from 0 to 14.4%) clearly larger than the defined threshold. Therefore, the vouchers of the species await to be checked to resolve the problem.

**Table 1.** GenBank accession data used in the analysis; * data from this study.

Species	Sample ID	GenBank Accession	Species	Sample ID	GenBank Accession
*Stenochironomusannulus*	ZJCH220, *	ON002477	*Stenochironomuslinanensis*	ZJCH251, *	ON002473
*Stenochironomusannulus*	ZJCH222, *	ON002475	*Stenochironomusokiabbus*	ZJCH219, *	ON002483
*Stenochironomusannulus*	ZJCH223, *	ON002480	*Stenochironomusokiabbus*	ZJCH244, *	ON002479
*Stenochironomusbaishanzuensis*	ZJCH246, *	ON002482	*Stenochironomusokiabbus*	ZJCH245, *	ON002474
*Stenochironomusbaishanzuensis*	ZJCH247, *	ON002476	*Stenochironomusokiabbus*	NC_061972	NC_061972
*Stenochironomusbaishanzuensis*	ZJCH226, *	ON002484	*Stenochironomusokiabbus*	NIESH0017	LC462301
*Stenochironomusfascipennis*	ZMUO.025362	MZ657887	*Stenochironomusokiabbus*	NIESH0026	LC462365
*Stenochironomusfascipennis*	ZMUO.025363	MZ658663	*Stenochironomusokiabbus*	NIESH0307	LC462355
*Stenochironomusgibbus*	JN016848	JN016848	*Stenochironomusokiabbus*	NIESH0746	LC462364
*Stenochironomusgibbus*	NC_061971	NC_061971	*Stenochironomusokiabbus*	OL753645	OL753645
*Stenochironomusgibbus*	OL742440	OL742440	*Stenochironomus* sp. 1CZ	OL753646	OL753646
*Stenochironomusgibbus*	PY-32A	KP902798	*Stenochironomus* sp. 2CZ	OL742441	OL742441
*Stenochironomusgibbus*	PY-33A	KP902799	*Stenochironomus* sp. 3CZ	OL753647	OL753647
*Stenochironomusgibbus*	PY-34A	KP902800	*Stenochironomus* sp.1BD	HQ551963	HQ551963
*Stenochironomusgibbus*	STE-GIB-IM-LIM28VII-254	MT535051	*Stenochironomus* sp.1BD	HQ928366	HQ928366
*Stenochironomusgibbus*	STE-HIB-IM-VI29VII-304	MT535060	*Stenochironomus* sp.1BD	MF718872	MF718872
*Stenochironomusgibbus*	ZMUO.024239	MZ657365	*Stenochironomus* sp.1BD	MF721507	MF721507
*Stenochironomusgibbus*	ZMUO.024238	MZ656608	*Stenochironomus* sp.1BD	MF723892	MF723892
*Stenochironomushibernicus*	ZMUO.025366	MZ660623	*Stenochironomustobaduodecimus*	NC_061973	NC_061973
*Stenochironomushibernicus*	ZMUO.025367	MZ656796	*Stenochironomustobaduodecimus*	OL753648	OL753648
*Stenochironomuslinanensis*	ZJCH224, *	ON002473	*Stenochironomuszhengi*	NC_061974	NC_061974
*Stenochironomuslinanensis*	ZJCH249, *	ON002478	*Stenochironomuszhengi*	OL753649	OL753649

**Table 2.** Kimura 2-parameter pairwise genetic distances based on COI barcodes of twelve known *Stenochironomus* species from GenBank.

Species	Distance										
	1	2	3	4	5	6	7	8	9	10	11
1. *S.annulus*											
2. *S.baishanzuensis*	15.13										
3. *S.fascipennis*	13.74	14.00									
4. *S.gibbus*	11.17	15.58	15.51								
5. *S.hibernicus*	16.50	15.36	14.39	14.61							
6. *S.linanensis*	17.74	18.69	18.29	17.04	18.22						
7. *S.okiabbus*	15.25	15.03	16.20	15.49	15.66	17.69					
8. *Stenochironomus* sp.1BD	14.92	14.28	13.91	15.26	15.35	15.92	14.27				
9. *Stenochironomus* sp.1CZ	18.58	17.11	17.16	18.03	21.01	19.58	19.08	15.80			
10. *Stenochironomus* sp.3CZ	12.87	14.98	14.20	14.83	17.19	17.69	14.89	14.46	17.39		
11. *S.tobaduodecimus*	18.11	17.05	18.11	18.32	19.08	19.04	16.12	20.12	17.40	18.83	
12. *S.zhengi*	17.96	16.59	18.15	19.14	19.32	19.65	18.16	18.43	20.55	17.70	20.09

### ﻿Taxonomy

#### ﻿ Stenochironomus annulus

Taxon classificationAnimaliaDipteraChironomidae

Song & Qi sp. nov.

168CDC8D-9D95-5473-AFC8-E696391A6376

http://zoobank.org/793EEC94-E1E4-45F7-906E-13154716B102

Figs 3 , 4 , 5

##### Type material.

***Holotype*** (BOLD & TZU sample ID: ZJCH220; Field ID: BSZ87) 1 male, China, Zhejiang Province, Lishui City, Qingyuan County, Baishanzu National Nature Reserve, 27.76°N, 119.31°E, 11–12. VIII. 2020, light trap, Qi X & Song C. ***Paratypes***: 2 males, same data as for holotype.

##### Diagnostic characters.

The adult males of *S.annulus* sp. nov. can be separated from known *Stenochironomus* species from China by the following combination of characters: spots on the membrane of wing restricted to RM and FCu areas; posterior portion of median vittae with little pale pigmentation; lateral vittae with stripe markings; postnotum with markings reaching the posterior margin; femur of mid and hind legs with an annulus medially on each; superior volsella cylindrical, with four long setae; inferior volsella extending beyond apex of anal point, with two or three bristles and one well-developed terminal spine.

##### Etymology.

The specific name refers to the circular ring markings of the femur of mid and hind legs of the male adult.

##### Description.

Male imago (N = 3). Total length 3.59–4.17, 3.87 mm. Wing length 1.95–2.03, 2.00 mm. Total length / wing length 1.75–2.05, 1.94. Wing length / length of pro-femur 1.58–1.93, 1.73.

**Figure 3.**
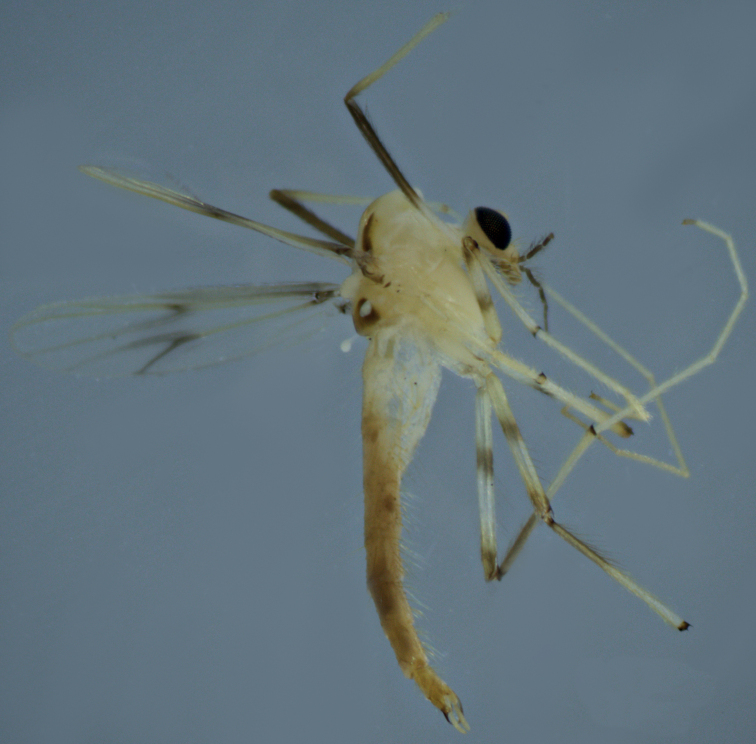
Male adult (holotype, in lateral view) of *Stenochironomusannulus* Song & Qi sp. nov.

***Coloration*** (Fig. 3). Mature adult mostly brownish. Head yellow. Thorax yellowish except for the lateral vittae, postnotum with dark pigmentation and medial lateral with light pigmentation (sometimes difficult to observe). Membrane with 2 dark spots restricted to RM and FCu areas. Legs. Apical 3/5 of femur of P1 dark brown; apical and annulus of femur, and Ta 5 P2–P3 dark brown; basal 1/2 of tibia of P3 dark brown. Abdomen. Tergites V–IX brown.

***Head*.**AR 1.62–1.88 (2), ultimate flagellomere 680–770 µm long; Temporals 10–13, 12 setae including 5–8 inner, and 2–3 outer, verticals, postorbitals 1–3. Clypeus with 15–22, 19 setae. Tentorium 170–205 μm long, 45–53 μm wide at the widest part. Palp 5-segmented, lengths (in μm) of segments: 60–70, 66; 50–70, 60; 193–220, 203; 140–160, 152; 208–288, 248. Palpomere ratio (5^th^ / 3^rd^) 1.09–1.31, 1.22.

***Thorax*.** Dorsocentrals 16–17, 18; acrostichals 12–15, 14; prealars 5–6, 6; Scutellum with 11–13 setae in 2 rows.

**Figure 4.**
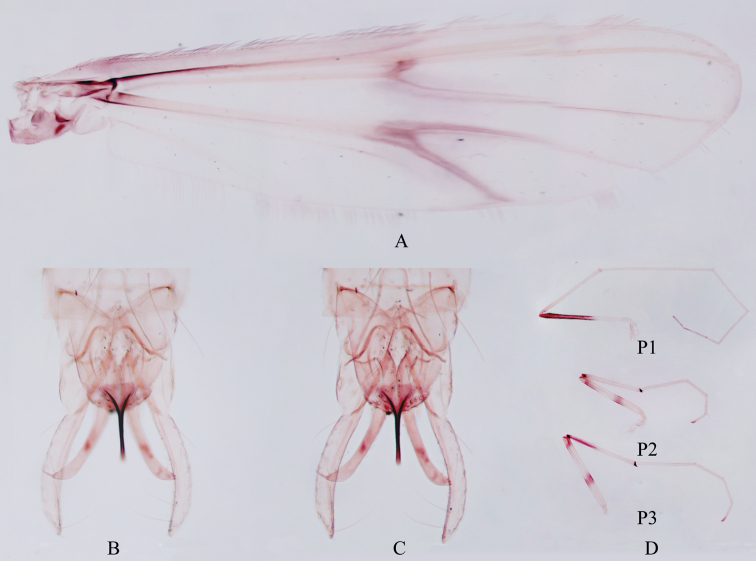
Male adult of *Stenochironomusannulus* Song & Qi, sp. nov. **A** wing **B** hypopygium in dorsal view **C** hypopygium in ventral view **D** legs.

***Wing*** (Fig. 4A). VR 1.08–1.08, 1.06. Brachiolum with 2–3 setae. Distribution of setae on veins: R, 31–39, 34; R1, 28–40, 34; R4+5, 33–62, 46. Squama with 8–12, 10 setae. Anal lobe normally developed.

***Legs*** (Fig. 4D). Fore leg: width at apex of tibia 43–50, 46 μm, tibia with blunt scale 35–40, 38 µm long. Mid leg: width at apex of tibia 53–65, 59 μm, tibia with 2 apical spurs 35–38, 37 and 40–45, 43 µm long. Hind leg: tibia 60–73 μm width at apex; tibial spurs 40–43, 42 and 40–43, 42 µm long, slightly fused medially. Lengths (in μm) and proportions of legs in Table 3.

**Table 3.** Male adult of *Stenochironomusannulus* sp. nov. Length (in µm) and proportions of legs (*N* = 3).

	P1	P2	P3
Fe	1060–1280, 1156	920–1110, 993	1120–1250, 1170
Ti	1050–1150, 1116	850–930, 893	1070–1100, 1060
Ta_1_	1500–1700, 1610	540–720, 656	860–910, 890
Ta_2_	750–820, 796	370–380, 373	460–500, 483
Ta_3_	630–660, 647	270–280, 273	360–380, 370
Ta_4_	440–570, 507	150–160, 156	220–220, 220
Ta_5_	210–240, 230	75–90, 82	80–100, 90
LR	1.42–1.48, 1.44	0.64–0.80, 0.73	0.82–0.85, 0.84
BV	1.75–1.80, 1.78	2.65–3.10, 2.87	2.64–2.78, 2.68
SV	1.35–1.45, 1.41	2.52–3.33, 2.91	2.42–2.61, 2.50

**Figure 5.**
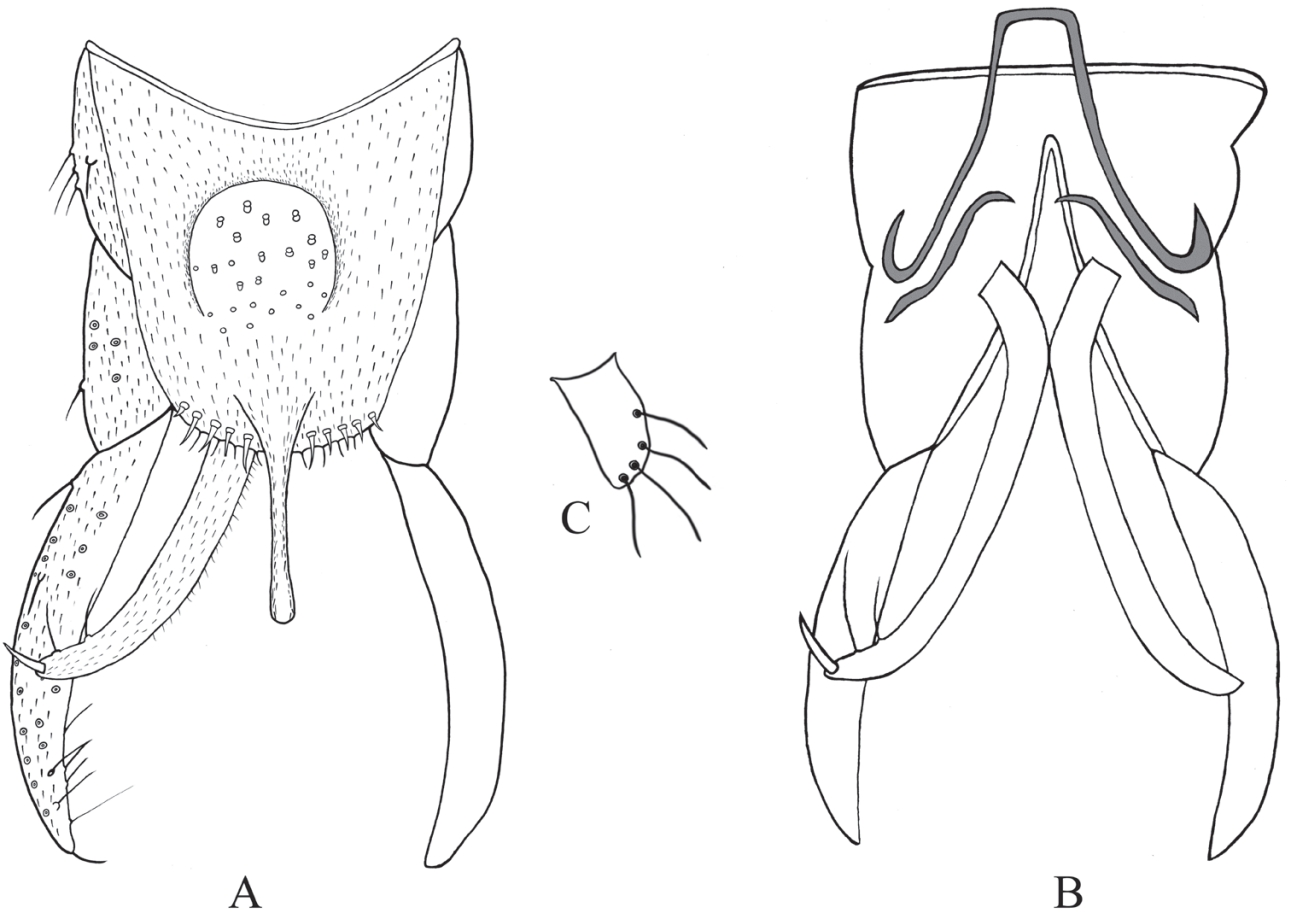
Male adult (holotype) of *Stenochironomusannulus* Song & Qi sp. nov. **A** hypopygium in dorsal view **B** hypopygium in ventral view **C** superior volsella.

***Hypopygium*** (Figs 4B, C, 5). Anal point straight and parallel-sided in dorsal view, 103–113, 100 μm long and 30–43, 35 µm wide at base, 8–10, 9 µm wide at apex. Tergite IX with 19–22, 20 long setae medially and posterior margin of tergite IX with 6 strong setae and 5 spines. Laterosternite IX with 4–4, 4 setae. Transverse sternapodeme 43–50, 47 μm long; phallapodeme 78–88, 85 μm long. Gonocoxite 173–185, 181 μm long, gonostylus 205–270, 238 μm long. Superior volsella cylindrical, 40–45, 43 μm long, 20–20, 20 μm wide, with 4–5 long setae (Fig. 4C). Inferior volsella elongate, 203–228 μm long, extending beyond the apex of anal point, with 2–3 long bristles and 1 strong terminal spine. HR 0.64–0.90, 0.77, HV 1.46–1.75, 1.64.

Immature stages and female unknown.

##### Remarks.

Morphologically, *S.annulus* sp. nov. shows high similarity to *Stenochironomusxianjuensis* Zhang, Gu, Qi & Wang, 2016, on the basis of the following similar common characters: membrane of wing with similar spot patterns; cylindrical superior and inferior volsella. However, the new described species could be distinguished in having a straight and parallel-sided anal point and different leg pigmentation patterns. According to the molecular data, *S.annulus* is sister to *S.gibbus* (Fig. 1), but could be separated by thorax vittate and leg coloration (Table 4).

**Table 4.** Main differences between *S.annulus* sp. nov., *S.baishanzuensis* sp. nov., *S.gibbus*, and *S.xianjuensis*.

	Thorax vittae	Anal point	Legs pattern
*S.annulus*	Median vittae not obvious; lateral vittae with stripe pigmentation	Anal point straight and parallel-sided	With dark annulus on femur of P2 and P3
*S.baishanzuensis*	Median vittae with little pigmentation; lateral vittae with stripe pigmentation	Anal point straight and parallel-sided	Entire femur of P2 pale; femur of P3 brown
*S.gibbus*	Thorax without pigmentation	Apex parallel-sided to slightly bulbous	Nearly 1/2 to entire femur of P2; basal 0.12–0.30 femur of P3 dark brown;
*S.xianjuensis*	Thorax without median vittae; lateral vittae with stripe pigmentation	Apex of anal point swollen and rounded	Apical 1/4 of P2 and P3 brown

##### Distribution.

The species is currently known only from Zhejiang Province in Oriental China.

#### ﻿ Stenochironomus baishanzuensis

Taxon classificationAnimaliaDipteraChironomidae

Song & Qi sp. nov.

6DA95692-6B0B-5822-962C-8C6E5694AC2C

http://zoobank.org/16D79540-7339-434A-9662-29D1BB5F360B

Figs 6 , 7 , 8

##### Type material.

***Holotype*** (BOLD & TZU sample ID: ZJCH226; Field ID: BSZ93) 1 male, China, Zhejiang Province, Lishui City, Qingyuan County, Baishanzu National Nature Reserve, 27.76°N, 119.31°E, 11–12. VIII. 2020, Qi X. & Song C., collected by light trap. ***Paratypes***: 2 males, same data as for holotype.

##### Diagnostic characters.

Adult males of *S.baishanzuensis* sp. nov. can be distinguished from other related species by the following combination of characters: membrane of wing with large dark spots on median and apical parts; median vitta, lateral vitta, and postnotum with pigmentation; superior volsella short and broad with four setae; inferior volsella with four long bristles and one stout terminal spine, not overreaching apex of anal point.

##### Etymology.

The specific name refers to the Baishanzu National Nature Reserve, where the holotype was collected.

##### Description.

Male imago (N = 3). Total length 3.98–4.22, 4.07 mm. Wing length 1.90–2.13, 2.05 mm. Total length / wing length 1.87–2.22, 1.99. Wing length / length of pro-femur 1.63–1.65, 1.64.

**Figure 6.**
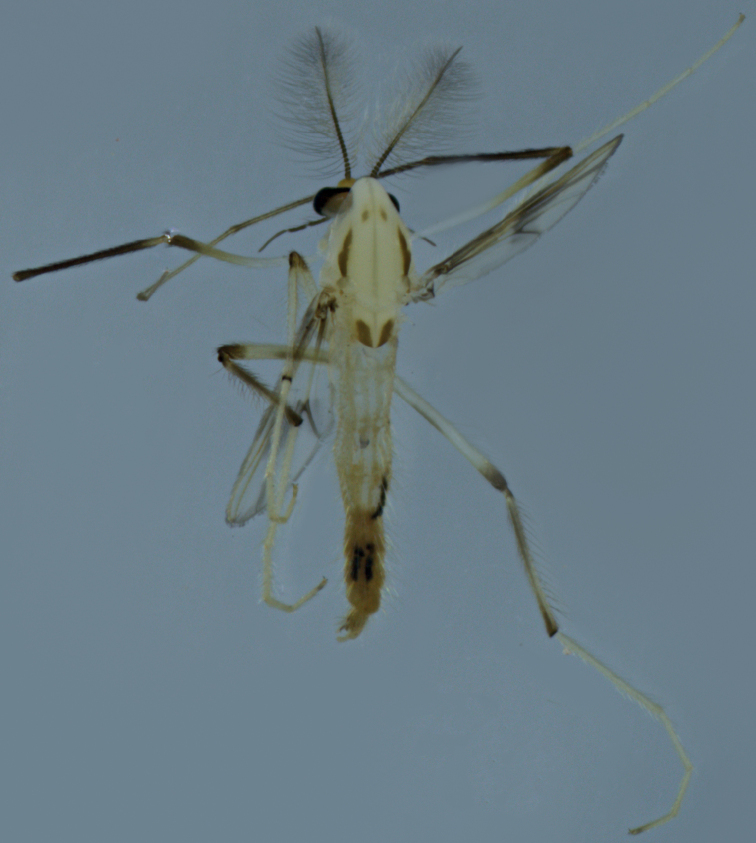
Male adult (holotype, in dorsal view) of *Stenochironomusbaishanzuensis* Song & Qi sp. nov. male.

***Coloration*** (Fig. 6). Head. Antennal hairs dark; palpomeres dark. Thorax almost pale yellow, with posterior portion of media vitta, posterior 3/4 portion of postnotum, and almost the lateral vitta dark brown. Membrane with 2 dark spots located around RM and FCu areas (median spot is darker). Legs. P1: knee, tibia and Ta 1 dark brown, basal 3/4 of femur and Ta 2–5 pale yellowish; P2: yellowish with brownish knee; P3: yellowish with brownish knees and tibia. Abdomen almost light brown with T VI–VII dark brown.

***Head*.**AR 1.93, ultimate flagellomere 810 µm long (n = 1); Temporals 12–15, 13 setae, including 5–7, 6 inner and 4–6, 5 outer verticals and 2–3, 3, postorbitals. Clypeus with 14–20, 18 setae. Tentorium 155–193, 170 μm long, 35–53, 44 μm wide. Palp 5-segmented, lengths (in μm) of segments: 42–55, 47; 43–65, 53; 200–223, 214; 130–155, 146; 232–275, 253. Palpomere ratio (5^th^ / 3^rd^) 1.13–1.26.

***Thorax*.** Dorsocentrals 15–16, 15; acrostichals 12–15, 14; prealars 6–7, 7. Scutellum with 8–9, 9 setae in 2 rows.

**Figure 7.**
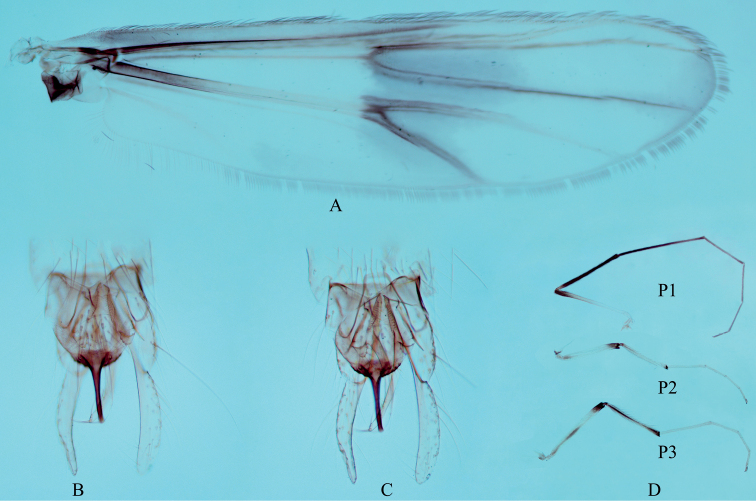
Male adult (holotype) of *Stenochironomusbaishanzuensis* Song & Qi, sp. nov. **A** wing **B** hypopygium in dorsal **C** hypopygium in ventral view **D** legs.

***Wing*** (Fig. 7A). VR 1.05–1.06, 1.06. Brachiolum with 2 setae. Distribution of setae on veins: R, 30–32, 31; R1, 22–26, 25; R4+5, 36–42, 39. Squama with 10–11, 11 setae. Anal lobe normally developed.

***Legs*** (Fig. 7D). Fore leg: apex of tibia 55–61, 58 μm width, tibia with pointed scale 35–38 µm long. Mid leg: apex of tibia 60–68, 65 μm width; tibial spurs 48–55, 51 and 45–55, 49 µm long, completely fused at midline part. Hind leg: apex of tibia 60–68, 64 μm width, tibia with 2 apical spurs 37–50, 47 and 35–47, 41 µm long. Lengths (in μm) and proportions of legs in Table 5.

**Table 5.** Male adult of *Stenochironomusbaishanzuensis* sp. nov. Lengths (in µm) and proportions of legs (*N* = 3, except where otherwise stated).

	P1	P2	P3
Fe	1150–1300, 1250	970–1110, 1053	1140–1310, 1243
Ti	1130–1280, 1230	870–930, 907	1050–1250, 1160
Ta_1_	1250 (*N* = 1)	480–700, 606	820–950, 900
Ta_2_	800 (*N* = 1)	370–400, 387	450–520, 477
Ta_3_	680 (*N* = 1)	300–340, 317	380–410, 397
Ta_4_	560 (*N* = 1)	180–210, 200	230–260, 240
Ta_5_	270 (*N* = 1)	90–100, 95	90–110, 100
LR	0.98 (*N* = 1)	0.52–0.75, 0.67	0.76–0.78, 0.77
BV	2.35 (*N* = 1)	2.46–2.64, 2.57	2.59–2.94, 2.72
SV	2.06 (*N* = 1)	2.91–4.17, 3.31	2.66–2.67, 2.67

**Figure 8.**
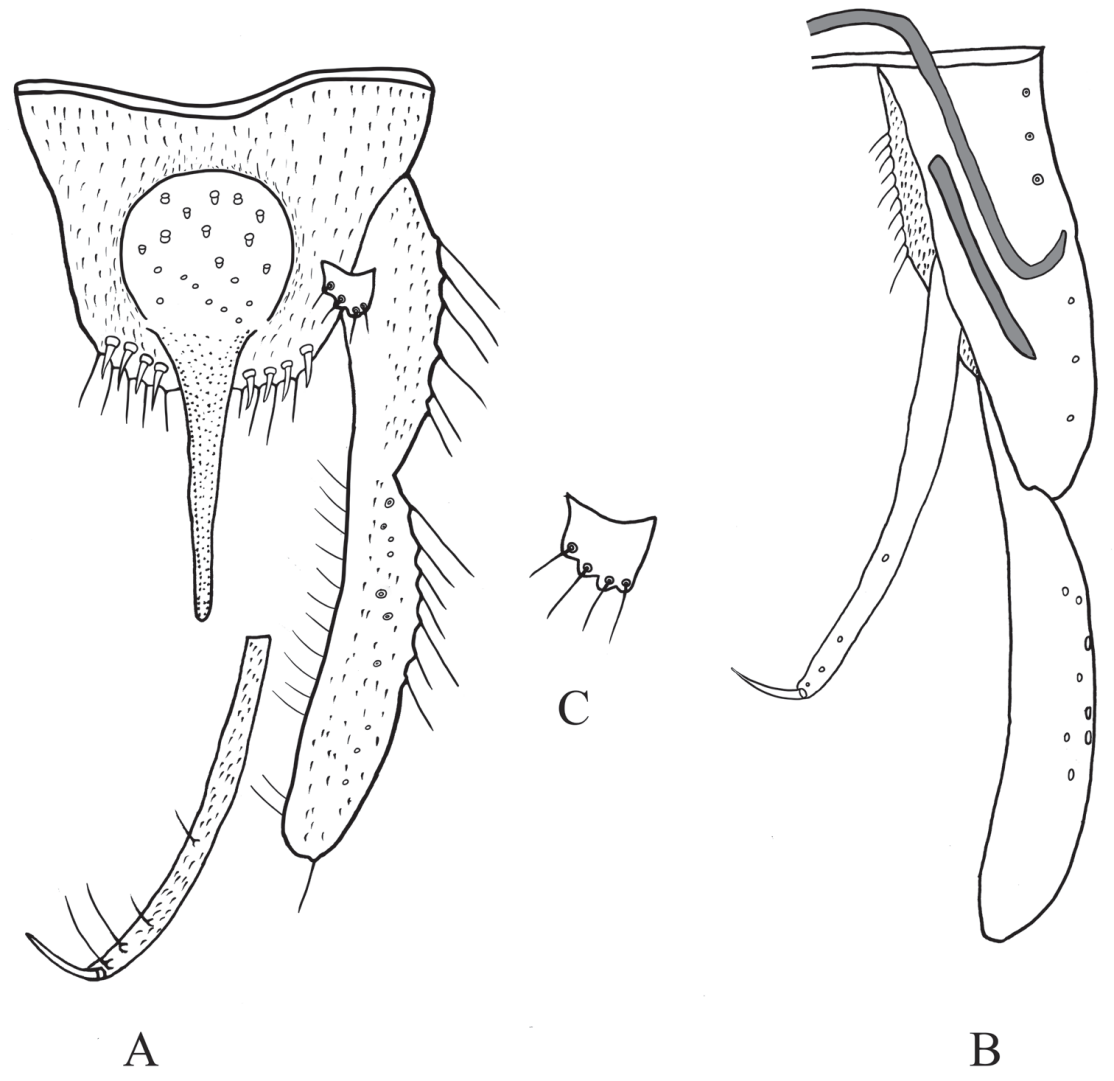
Male adult (holotype) of *Stenochironomusbaishanzuensis* Song & Qi, sp. nov. **A** hypopygium in dorsal view **B** hypopygium in ventral view **C** superior volsella.

***Hypopygium*** (Figs 7B, C, 8). Anal point 110–125, 120 μm long, 30–45, 35 µm wide at base, 10–13, 12 µm wide at apex. Tergite IX with 18–23, 20 long setae medially, and 3–4, 4 setae laterally. Posterior margin of tergite IX with 3–4, 4 spines and 5–7 long setae each side. Transverse sternapodeme 38–53, 44 μm long; phallapodeme 95–113, 105 μm long. Gonocoxite 183–188, 185 μm long. Gonostylus 218–25, 221 μm long. Superior volsella short and broad, 13–15, 14 μm long, 18–25, 21 μm wide, with 4 long setae (Fig. 7C). Inferior volsella elongate, 183–192, 187 μm long, extending at most, to apex of anal point, with 4 setae and 1 strong terminal spine. HR 0.83–0.84, .084, HV 1.77–1.93.

Immature stages and female unknown.

##### Remarks.

The male adult of *S.baishanzuensis* sp. nov. resembles that of *S.gibbus* (Fabricius, 1794) in the structure of the hypopygium and the wing patterns, but can be separated by the following characters: straight and parallel-sided anal point, and legs bearing different patterns (Table 4).

##### Distribution.

The species is currently known only from Zhejiang Province, Oriental China.

#### ﻿ Stenochironomus okialbus

Taxon classificationAnimaliaDipteraChironomidae

Sasa, 1990

E660BFC4-1161-56C1-9A67-A44404417C81

Figs 9 , 10

Stenochironomus okialbus Sasa, 1990: 122, fig. 10.

##### Material examined.

3 male adults, collected by light trap in Zhejiang Province, Lishui City, Qingyuan County, Baishanzu National Nature Reserve, 27.76°N, 119.31°E, 11–12.VIII.2020, leg. Song C.

**Figure 9.**
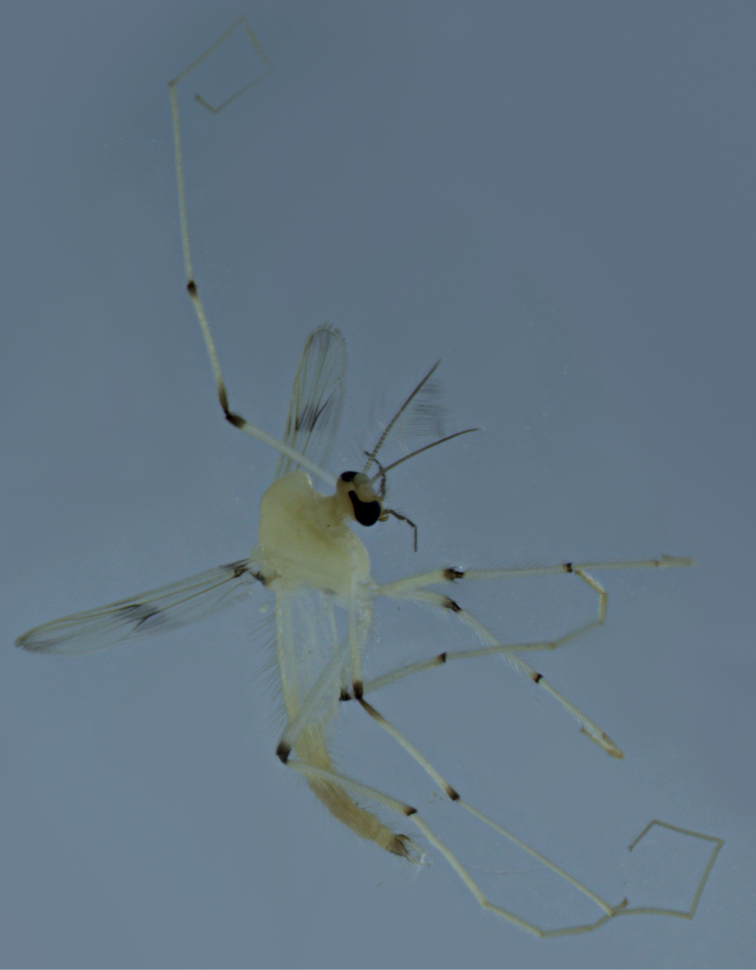
Male adult of *Stenochironomusokialbus* (in lateral view): new record to China

##### Diagnostic characters.

*Stenochironomusokialbus* differs from other related species by a combination of characters: wing with dark markings in the middle and apex; superior volsella short and small, spatulate, with four or five long setae; inferior volsella elongate, with 2–4 long setae and a slender terminal spine; posterior margin of tergite IX with 8–10 setae and eight spines.

##### Description.

Male imago (N = 3). Total length 2.94–3.98, 3.62 mm. Wing length 1.80–1.85 mm. Total length / wing length 1.85–2.20, Wing length / length of pro-femur 178–1.88.

***Coloration*** (Fig. 8). Body almost pale yellowish or white, except postnotum with spot area and tergite IV–VII brown; wing with dark pigmentation on median and apical parts; all legs pale yellow with dark knees and apex of tibia.

***Head.***AR 1.10–1.25, 1.12, ultimate flagellomere 430–660, 580 µm long. Temporal with 12–16, 14 setae, including 7–8, 7 inner verticals, 4–7, 6 outer verticals and 1–2, 2 postorbitals. Clypeus with 12–20, 17 setae. Tentorium 155–220, 190 μm long, 30–53, 44 μm wide. Palpomere lengths (μm): 33–48, 37; 40–65, 55; 123–195, 170; 85–125, 116; 130–218, 194. Palpomere ratio (5^th^ /3^rd^) 1.05–1.21, 1.13.

***Thorax.*** Dorsocentrals 14–21, 18; acrostichals 9–12, 11; prealars 5–6, 6; Scutellum with 7–12 setae in two rows.

***Wings*.**VR 1.06–1.10, 1.08. Brachiolum with 1–2, 2 setae. Distribution of setae on veins: R, 20–29, 25; R1, 21–39, 31; R4+5, 49–53, 51. Squama with 10–18, 14 setae. Anal lobe normally developed.

***Legs.*** Fore leg: width at apex of tibia 53–63, 58 μm, tibia with blunt scale 35–50, 44 µm long. Mid leg: width at apex of tibia 53–70, 61 μm, tibia with two apical spurs 43–53, 48 and 45–58, 52 µm long. Hind leg: width at apex of tibia 58–73, 64 μm, tibia with two apical spurs 45–55, 51 and 45–56, 52 µm long. Lengths (in μm) and proportions of legs as in Table 6.

**Table 6.** Male adult of *Stenochironomusokialbus*. Lengths (in µm) and proportions of legs (*N* = 3, except where otherwise stated).

	P1	P2	P3
Fe	920–1310, 1150	790–1220, 1053	930–1350, 1183
Ti	930–1210, 1050	730–1030, 913	870–1230, 1083
Ta_1_	1200–1640 (*N* = 2)	530–730, 653	710–990, 880
Ta_2_	590–810 (*N* = 2)	290–410, 360	380–520, 470
Ta_3_	500–680 (*N* = 2)	230–310, 277	290–410, 363
Ta_4_	450–650 (*N* = 2)	140–210, 180	180–250, 230
Ta_5_	210–300 (*N* = 2)	60–100, 87	80–110, 97
LR	1.45–1.48 (*N* = 2)	0.70–0.73, 0.72	0.80–0.82, 0.81
BV	1.63–1.69 (*N* = 2)	2.84–2.94, 2.90	2.69–2.75, 2.71
SV	1.42–1.46 (*N* = 2)	2.88–3.08, 3.00	2.53–2.61, 2.57

**Figure 10.**
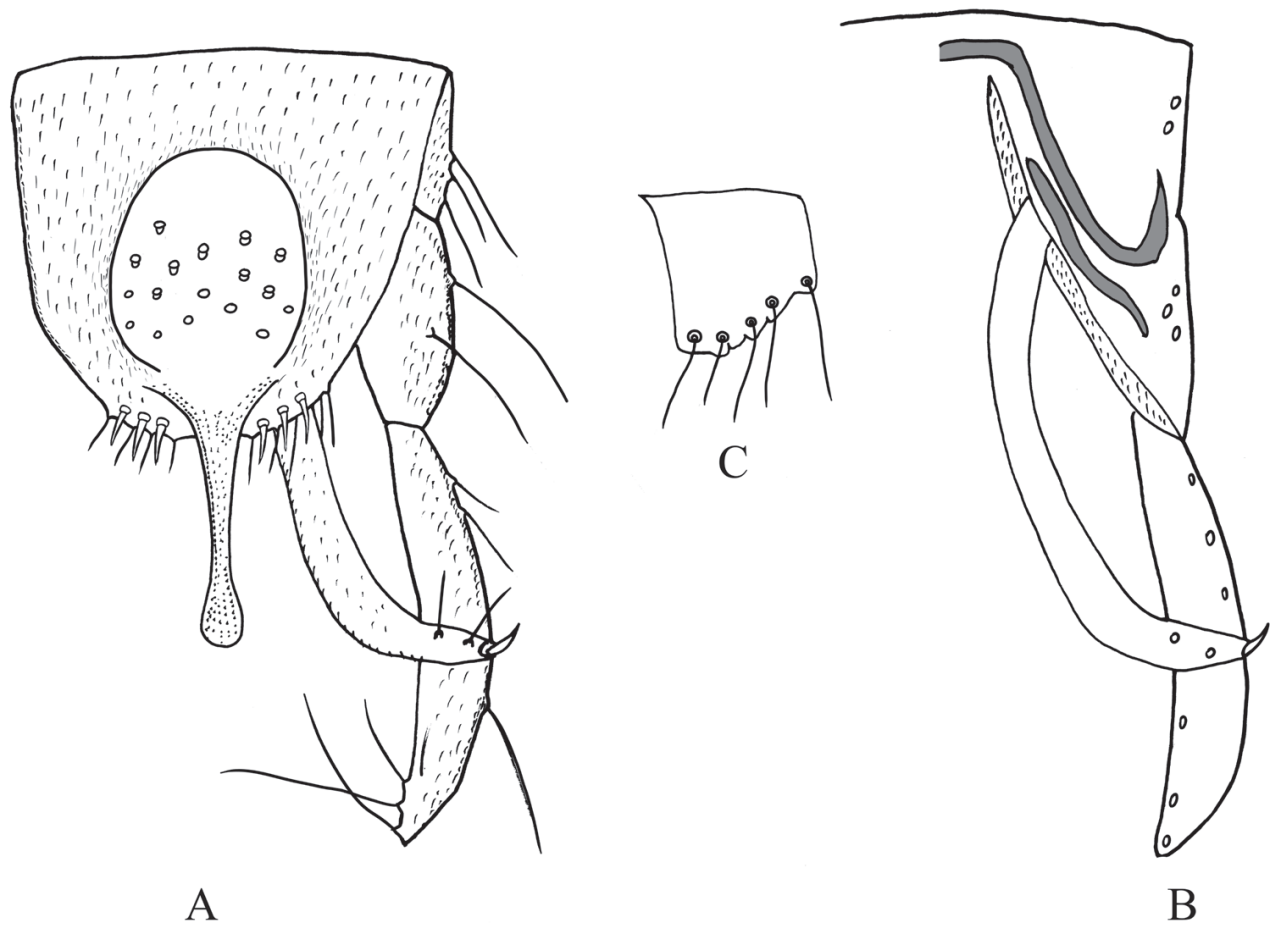
Male adult of *Stenochironomusokialbus*. **A** hypopygium in dorsal view **B** hypopygium in ventral view **C** superior volsella.

***Hypopygium*** (Fig. 10). Anal point 110–125, 118 μm long, 43–60; wide, 52 µm width at base, 18–20, 18 µm at apex; slightly swollen and rounded apically. Tergite IX with 19–25, 22 long setae on median part; posterior margin of tergite IX with 6 strong setae and 3 spines. Laterosternite IX with 3–4, 4 setae. Transverse sternapodeme 35–48, 41 μm long; phallapodeme 85–95, 89 μm long. Gonocoxite 138–175, 159 μm long. Gonostylus 185–200, 196 μm long. Superior volsella short, 25–30, 28 μm long, 23–25, 24 μm wide, with 4–5 long setae. Inferior volsella linearly elongate, 183–220, 208 μm long, with 2–3 long setae and 1 stout terminal spine. HR 0.74–0.86, 0.81; HV 1.58–1.97, 1.84.

##### Remarks.

The morphological characters of the Chinese specimens fit well with the original description and illustrations provided by Sasa (1990): wing with two spotted areas; narrow dark rings on knee points; anal point slightly swollen and rounded. However, some relevant differentiating characters were observed within the examined specimens: the inferior volsella have only two or three long setae, while it bears four in the Japanese specimens; average values of the AR 1.10–1.25 are lower than 1.37–1.41 in the Japanese species.

According to the molecular data, specimen (LC462365) of *Stenochironomusokialbus*, shows a large genetic distance to other specimens (up to 14%); as the specimen is not accessible it should be rechecked. The K2P distance between Japanese and Chinese specimens is 1.7%, which well supports them as the same species.

##### Distribution.

Oriental China (Zhejiang) and Japan.

#### ﻿ Stenochironomus linanensis

Taxon classificationAnimaliaDipteraChironomidae

Qi, Lin, Liu & Wang

279D2BA2-9200-54DF-A2F6-092DFDB23967

Stenochironomus linanensis Qi et al. (2015): 114.

##### Material examined.

3 male adults collected by light trap, leg. Qi X.; Zhejiang Province, Lishui City, Qingyuan County, Baishanzu National Nature Reserve, 27.76°N, 119.31°E, 11–12.VIII.2020.

##### Diagnostic characters.

*Stenochironomuslinanensis* differs from other related species in having: wing transparent; body yellow; superior volsella finger-like with nine long setae; inferior volsella elongate, with four long setae and one strong terminal spine; tergite IX with 10–15 long setae located medially.

##### Distribution.

Oriental China (Zhejiang).

#### ﻿ Stenochironomus satorui

Taxon classificationAnimaliaDipteraChironomidae

(Tokunaga & Kuroda, 1936)

13FC47D7-A5D7-56F4-81A3-A680CFACB207

Chironomus (Stenochironomus) satorui Tokunaga & Kuroda (1936): 2.
Stenochironomus satorui Qi et al. (2011): 226.

##### Material examined.

2 male adults collected by light trap, leg. Song C., Zhejiang Province, Lishui City, Qingyuan County, Baishanzu National Nature Reserve, 27.76°N, 119.31°E, 11–12.VIII.2020.

##### Diagnostic characters.

Wing with median band; posterior edge of tergite IX with 14–15 long setae; anal point slender parallel-sided, with pointed apex; superior volsella short and finger-like, with four or five setae; inferior volsella with one median seta and three apical setae.

##### Distribution.

China (Zhejiang, Hainan, Guizhou, Xizang); Japan.

### ﻿Updated key to known adult males of *Stenochironomus* from China

The following key updates Lin et al. (2021) and Qi et al. (2015)

**Table:** 

1	Inferior volsella with a well-developed terminal spine	**2**
–	Inferior volsella without a well-developed terminal spine	**12**
2	Wing membranes with dark pigmentation	**3**
–	Wing membranes without any pigmentation	**8**
3	Legs almost entire brown; wing with dark pigment restricted to a part area	**4**
–	Legs yellow; entire wing smoky gray	***S.maculatus* Borkent, 1984**
4	Wing with two dark spots restricted to RM and FCu areas	**5**
–	Wing with dark median band	**6**
5	Anal point straight and parallel-sided	***S.annulus* Song & Qi, sp. nov.**
–	Apex of anal point swollen and rounded	***S.xianjuensis* Zhang et al., 2016**
6	Anal point bullous, knees of fore femur dark brown	**7**
–	Anal point almost parallel-sided, fore femur dark brown	***S.baishanzuensis* Song & Qi, sp. nov.**
7	Mid and hind legs without pigmentation except knees	***S.okialbus* Sasa, 1990**
–	Apical 0.23 to entire hind-femur with dark pigmentation	***S.gibbus* (Fabricius, 1805)**
8	Apex of anal point swollen and rounded	**9**
–	Apex of anal point not swollen and rounded	**11**
9	Superior volsella with 9–12 setae; posterior margin of tergite IX with 10–14 setae and 4–8 spines	**10**
–	Superior volsella with four setae; posterior margin of tergite IX with 14–16 setae	***S.koreanus* Borkent, 1984**
10	Superior volsella much beyond posterior margin of tergite IX; anal lobe reduced	***S.zhengi* Lin & Liu, 2021**
–	Superior volsella small, finger-like; anal lobe developed	***S.linanensis* Qi, Lin, Liu & Wang, 2015**
11	Posterior edge of tergite IX with eight long setae and six spines; anal point parallel-sided	***S.macateei* (Malloch, 1915)**
–	Posterior edge of tergite IX with 14 long setae, without any spine; anal point roughly triangular, apically pointed	***S.mucronatus* Qi, Shi & Wang, 2008**
12	Wing membranes with dark pigmentation	**13**
–	Wings without any pigmentation or with narrow pigment areas around RM and along veins M_3+4_ and Cu_1_ 12	**15**
13	Abdominal tergites I–IV light yellow, tergites V–VIII light brown, hypopygium dark brown	**14**
–	Abdomen and hypopygium light yellow	***S.inalemeus* Sasa, 2001**
14	Preepisternum with brown spots; anal point apically rounded	***S.nubilipennis* Yamamoto, 1981**
–	Preepisternum without any pigmentation; anal point apically pointed	***S.satorui* (Tokunaga & Kuroda, 1936)**
15	Posterior margin of tergite IX without spines	**16**
–	Posterior margin of tergite IX with spines	***S.brevissimus* Qi, Lin, Liu & Wang, 2015**
16	Entire body yellow, without dark pigmentation; inferior volsella with three long setae	***S.hainanus* Qi, Shi & Wang, 2008**
–	Body yellow, with brown spots on thorax, abdomen, hypopygium and legs; inferior volsella with six long setae	***S.totifuscus* Sublette, 1960**

## Supplementary Material

XML Treatment for Stenochironomus annulus

XML Treatment for Stenochironomus baishanzuensis

XML Treatment for Stenochironomus okialbus

XML Treatment for Stenochironomus linanensis

XML Treatment for Stenochironomus satorui
